# 73 Joint pains revealing a dysautonomic syndrome

**DOI:** 10.1093/rheumatology/keac496.069

**Published:** 2022-10-07

**Authors:** N Boutrid, H Rahmoune, H Boutrid, B Bioud, M Amrane, A.S Chehad

**Affiliations:** 1 LMCVGN Laboratory, Faculty of Medicine, Sétif-1 University, Algeria; 2 Pediatrics, Setif University Hospital, Algeria; 3 Faculty of Medicine, Algiers-1 University, Algeria; 4 Faculty of Medicine, Constantine-3 University, Algeria

## Abstract

**Background:**

Primary dysautonomia is a disorder of the autonomic nervous system with nonspecific manifestations, mainly affecting blood pressure and heart rate.

**Objective:**

We report the diagnostic odyssey in an 11-year-old girl starting with arthralgia and leading to the final diagnosis of a rare dysautonomia.

**Methods:**

A 13-year-old girl, with no pathologic history, was admitted to pediatrics for diffuse arthralgia without clinical inflammatory signs.

Biological exploration unmasked a morning proteinuria without hypoalbuminemia and with a normal electrophoretic profile, microcytic anaemia and orthostatic hypotension authenticated during his hospitalization.

Exhaustive explorations were undertaken (including autoimmune panel, ophthalomological examination, full metabolic screen and even renal biopsy), without any conclusive result.

**Results:**

The development—after one year of follow up—of arterial hypertension, associated with orthostatic hypotension and proteinuria, points toward the extremely rare defect in baroreceptors.

The management, in collaboration with the cardiology department, enhanced the patient’s quality of life with a reduction in hypertensive peaks. An appropriate lifestyle also amended the intensity of orthostatic hypotension.

The close multidisciplinary follow-up over >48 months is reassuring.

**Conclusion:**

The peculiar baroreceptor defect in a context of dysautonomia should be kept in mind in the (long) list of differential diagnosis of arthralgias, as joint pain can be triggered by dysautonomia. Management is mainly symptomatic.

